# P-994. Building a Culture of Stewardship: The Role of Infectious Diseases Pharmacists in Promoting Documentation Across Pharmacy Teams

**DOI:** 10.1093/ofid/ofaf695.1193

**Published:** 2026-01-11

**Authors:** Blain Thayer, Taylor Steuber, Taylor Nelson, Mary Reese, Andres Acevedo Bran

**Affiliations:** University of New Mexico Hospital, ALBUQUERQUE, NM; UMKC School of Pharmacy, Columbia, Missouri; University of Missouri- Columbia, Columbia, Missouri; Maury Regional Medical Center, Columbia, Tennessee; University of Missouri Health, Columbia, Missouri

## Abstract

**Background:**

Antimicrobial stewardship (AS) initiatives are critical for combating antibiotic resistance, improving patient outcomes, and ensuring optimal use of antimicrobials. Despite national guidelines and regulatory support, consistent documentation of AS interventions across pharmacy teams remains a challenge. This study evaluates the impact of integrating an infectious diseases (ID) pharmacist into a pharmacy team on AS documentation practices in a large academic medical center.

**Methods:**

This quasi-experimental pre-post study was conducted at University of Missouri Health, a 709-bed tertiary care academic hospital. The intervention involved onboarding a dedicated ID/ASP pharmacist in August 2023. Documentation of AS interventions was tracked using the Cerner electronic medical record and Power BI analytics from January 2023 to December 2024. Intervention volumes, types, and pharmacist contributions were analyzed using Mann-Whitney U and chi-square tests.

**Results:**

Following the addition of the ID/ASP pharmacist, the average number of monthly documented interventions increased from 152.4 ± 52.2 to 387 ± 122 (p < 0.00001). The ID/ASP pharmacist independently contributed an average of 104.3 ± 30.6 interventions monthly. The types of interventions shifted significantly, with the ID pharmacist more frequently documenting high-impact activities such as de-escalation (p < 0.00001) and escalation (p < 0.00001), compared to general pharmacists who primarily documented dose adjustments and communication-related activities.

**Conclusion:**

The integration of an ID/ASP pharmacist into the pharmacy team led to substantial improvements in both the frequency and complexity of documented interventions. These findings highlight the importance of pharmacist-driven stewardship and underscore how focused documentation efforts can foster a culture of accountability and clinical excellence in AS programs.

**Disclosures:**

Blain Thayer, PharmD, BCIDP, AAHIVP, AbbVie Inc: Honoraria

